# Voltage-Tunable Nonlocal Metasurface for Enhanced Outcoupling of Emission from Quantum Dots

**DOI:** 10.1021/acs.nanolett.5c04834

**Published:** 2026-01-20

**Authors:** Samuel Prescott, Prasad P. Iyer, Sanghyeok Park, Stephanie Malek, Jiho Noh, Pingping Chen, Chloe F. Doiron, Sadhvikas Addamane, Igal Brener, Oleg Mitrofanov

**Affiliations:** † 4919 University College London, Electronic and Electrical Engineering, London WC1E 7JE, U.K.; ‡ Center for Integrated Nanotechnologies, 1105 Sandia National Laboratories, Albuquerque, New Mexico 87123, United States; § 1105 Sandia National Laboratories, Albuquerque, New Mexico 87123, United States; ∥ 1877 University of Colorado Boulder, Electrical, Computer and Energy Engineering, Boulder, Colorado 80309, United States

**Keywords:** Single-photon source, nonlocal metasurface, quantum information, quantum dot, quantum-confined Stark effect, emission enhancement

## Abstract

Cooperative emission of indistinguishable photons from multiple distant sources can enable quantum information processing, and low-density semiconductor quantum dots (QDs) embedded in metasurfaces hold promise to scale up this functionality. However, the inhomogeneity in size within QD ensembles and limited interresonator coupling in local metasurfaces make this effect highly unlikely. Here, we demonstrate a nonlocal metasurface platform with embedded GaAs QDs coupled to extended photonic modes with emission wavelength tunability and enhanced free-space emission outcoupling. Natural variation in the QD dipole moment allows us to tune two QDs into spectral alignment and resonance with selected modes. As a result, two distant QDs can produce same-wavelength photons with strongly improved outcoupling efficiency to free space. The nonlocal and periodic nature of the developed metasurface eliminates the need for precise placement of individual QDs and, although cooperative emission was not yet demonstrated, this metasurface platform opens doors for investigations of cooperative effects for quantum information system applications.

## Graphical abstract

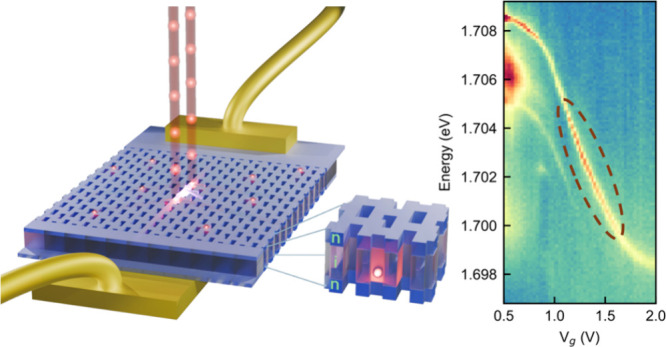

Cooperative emission of indistinguishable photons from multiple sources, or superradiance, is a fundamental quantum phenomenon^1–4^ and an enabling functionality for quantum communications and quantum information processing.^5^ It can be realized with identical emitters,^6^ such as atomic defects in crystals, placed within a subwavelength size volume^7^ or within a cavity^8,9^ or waveguide^6,10^ by exploiting a distributed photonic mode. While the effect requires emitters to be identical, semiconductor quantum dots (QDs) with emission wavelength tuning^11–19^ can serve as an attractive alternative to atomic defects, offering excellent single-photon emission properties^20,21^ with relatively high emission rates and wavelength selectivity.^20,22–25^ However, due to the large inhomogeneity in size within practical ensembles of QDs, this approach requires selected emitters to be placed with high precision within a photonic structure, such as waveguides^15,26–29^ and cavities,^30–32^ hindering scalability.^33–35^

Dielectric metasurfaces hold promise to circumvent this technologically challenging requirement owing to their periodic structure that mitigates the reliance on fabrication with intricate alignment.^36^ Furthermore, metasurfaces could potentially enable not only the coupling between individual QDs, but also outcoupling into free space,^37–41^ thus facilitating access to any QD within the metasurface. Mie metasurfaces with embedded QDs have already shown over an order-of-magnitude improvement in photon outcoupling efficiency.^40,41^ However, no superradiance has been observed so far, and this can be attributed to the challenge of finding spectrally overlapping multiple QDs and the local nature of Mie modes, which limits the capacity for coupling QDs distributed over distant resonators. Although evanescent coupling between neighboring Mie resonators exists, it is relatively weak, making cooperative emission from embedded QDs unlikely, especially without a provision for emission wavelength tuning.

In contrast, recently proposed nonlocal metasurfaces,^42–45^ designed to support spatially extended (or guided) and wavelength-selective optical resonances, could facilitate the coupling between distant QDs, while also enabling photon outcoupling to free space via breaking metasurface symmetries. In particular, nonlocal metasurfaces based on recently introduced dimers and quadromers can support multiple quasi-bound states in the continuum (q-BICs) and guided mode resonances,^46,47^ which can be robustly engineered for a desired wavelength, for example by exploiting Brillouin zone folding and symmetry breaking operations.^46^ Nonlocal metasurfaces have been demonstrated for emission control, wavefront shaping, thermal imaging and entangled photon pair generation.^48–54^ Here, we demonstrate a nonlocal metasurface platform with embedded GaAs QDs coupled to extended photonic modes with emission wavelength tunability and enhanced free-space outcoupling. We found that this platform allows two QDs to be tuned to emit photons at the same wavelength using an externally applied electric field, via the quantum confined Stark effect (QCSE),^16,18^ while also enhancing the outcoupling efficiency by over one order of magnitude. Notably, this is achieved without the need for individual QD alignments within the metasurface. Natural variation in the QD dipole moment permits the tuning of two QDs into a spectral alignment with each other. This has the potential to provide an on-chip voltage-controllable platform for cooperative emission from multiple sets of quantum emitters, enabling the development of scalable quantum communication and information systems.

We developed a metasurface platform for simultaneous emission from distant tunable epitaxially grown QDs (illustrated in Figure 1a). To do so, we considered QD growth, and heterostructure and metasurface designs for enabling tunability and enhanced photon outcoupling. We selected low-density local-droplet-etched (LDE) GaAs QDs, grown by molecular beam epitaxy.^22^ In previous studies, LDE GaAs QDs showed excellent single-photon emission characteristics, exhibiting a high degree of monodispersity^16,25,40^ and wavelength tunability;^24^ which are essential qualities for enabling cooperative emission.^17,21,55^ The QDs were embedded within a 90 nm thick Al_0.4_Ga_0.6_As barrier, with *n*-doped Al_0.15_Ga_0.85_As layers, each 45 nm thick, above and below the barrier (see the Supporting Information) to enable tuning.

**Figure 1:**
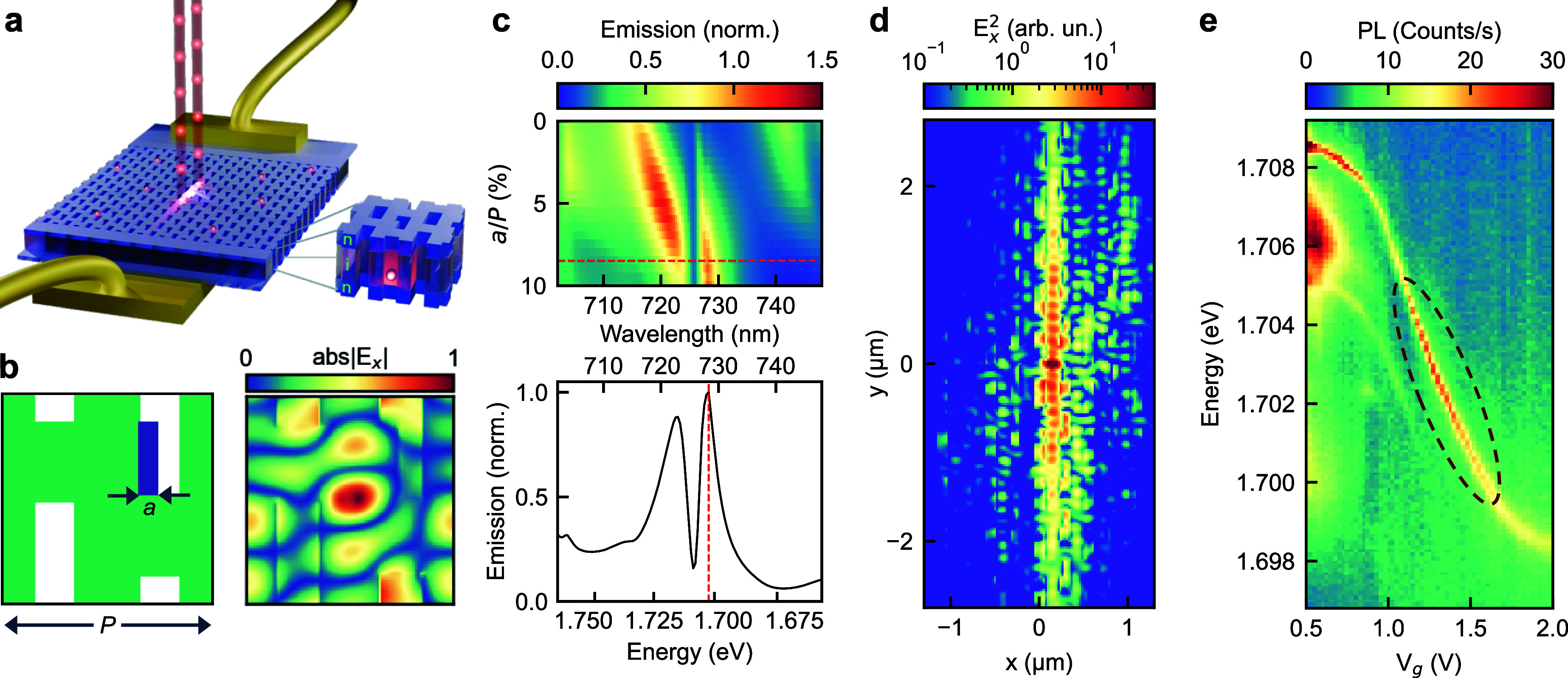
**a.** Illustration of the tunable metasurface platform with multiple QDs, embedded in the vertical *n-i-n*-Schottky diode structure. **b.** Metasurface unit cell (left) and electric field profile at a wavelength of 728 nm (1.703 eV) (right). **c.** Simulated map of outcoupling efficiency for a range of perturbations *a* (*upper panel*) and the outcoupling efficiency spectrum for the selected metasurface design (*lower panel*). The emission efficiency is normalized to its maximum at 728 nm (1.703 eV) for *a*/*P* = 0.085. **d.** Simulated optical field (*E*_*x*_^2^) due to an embedded *x*-polarized dipole emitter positioned at the metasurface center showed guided-mode behavior (at 728 nm). **e.** Photoluminescence (PL) map for a typical QD under applied bias ranging from 0.5 – 2.0 V showing enhanced emission in the circled region.

Using reactive ion etching, we isolated a small mesa and etched a 120 x 120 μm metasurface consisting of connected unit cells for electrical continuity. An Ohmic contact was formed at one edge of the mesa. Then, the sample was transferred onto a sapphire substrate, the original GaAs substrate was removed, and a Schottky contact was formed at the opposite edge of the mesa, such that external bias can be applied across the barrier over the entire metasurface area. The device is illustrated in Figure 1a, and fabrication details are provided in the Supporting Information.

We designed our metasurface not only to support QD tuning with external voltage but also to facilitate both the coupling of distant QDs to a common mode and the outcoupling of photons into free space. We started with a design containing a symmetric arrangement of two rectangular holes (a dimer metasurface). It supports multiple modes, including a narrow-linewidth *x*-polarized guided mode at 728 nm distributed between the long sides of the holes and a broader-linewidth radiative mode at a slightly different wavelength. We then translated the holes along the *y*-axis in opposite directions; this transformation perturbs the guided mode wavelength only minimally, while it brings the radiative mode wavelength closer to the guided mode wavelength. These two modes now show a remarkably good spatial overlap (Figure 1b and Supporting Information), although they are still separated spectrally (see Figure 1c, *upper panel*).

The guided mode wavelength can be tuned by using the *y*-axis period (*P*) as a parameter, whereas the radiative mode can be tuned further by introducing a perturbation *a* in one of the holes (Figure 1b). We align the two modes spectrally using the latter method, which does not affect the guided mode wavelength. The tuning is illustrated in Figure 1c (*upper panel*), where we plot a simulated photon outcoupling efficiency map (emission) for an *x*-polarized point dipole emitter placed at the center of the unit cell with the variable perturbation *a* (simulations details are provided in the Supporting Information). The two modes are clearly visible in the emission map exhibiting an anticrossing behavior with a distinctive ∼5 nm-wide feature showing enhanced emission at a wavelength of ∼728 nm (∼1.703 eV). We will use this emission enhancement feature to identify the coupling of QDs to the metasurface modes in the experiment.

In addition to the emission enhancement, we investigated the nonlocal characteristics of the metasurface at this wavelength. We simulated the field formed within the metasurface due to a point dipole (*E*_*x*_) placed at the center of a finite-area metasurface (11 × 11 unit cells). We observe a field distribution extended substantially beyond the unit cell along the *y*-axis, the direction of propagation for the *x*-polarized guided mode resonance. This suggests that the guided mode may be used to facilitate inter-QD coupling. This nonlocal metasurface, and specifically the combination of enhanced emission outcoupling and the in-plane guided mode, has potential to mitigate the high-precision alignment requirement of the QD in a cavity approach. The nonlocal metasurface combines the waveguiding properties that have been previously demonstrated to enable cooperative emission from QDs^15^ with the alignment-free emission outcoupling enhancement properties shown in Mie metasurfaces,^41^ promising a practical alignment-free platform for cooperative quantum emission investigations.

We realized QD energy tuning by applying an external voltage, *V*_g_, to the metasurface sample. Photoluminescence (PL) from individual QDs embedded in the metasurface under bias was characterized using nonresonant (above the barrier bandgap) CW excitation at 516 nm (2.40 eV), and Figure 1e shows a typical QD PL map (*x*-polarized component, H). At low applied voltages, *V*_g_, the QD emission spectrum remains largely unchanged. However, as the voltage is increased above ∼0.5 V, the exciton line shifts to lower energies while the intensity initially decreases showing a local minimum at *V*_g_ = 1 V. Then, as the exciton line tunes, the PL intensity increases reaching a maximum at *V*_g_ = ∼1.25 V (Figure 1e) and then drops at higher voltages. Typically, QD exciton lines in these metasurfaces exhibit tunability over a range of ∼10–12 meV, within which we observed noticeable variation in the exciton line intensity. For the QD shown in Figure 1e, the line can be tuned to ∼728 nm (1.703 eV), the wavelength at which we expect enhanced photon outcoupling (Figure 1c), and indeed, the PL map shows emission enhancement near this wavelength (dashed circle).

To further confirm the role of the metasurface in the emission enhancement, we characterized the QD exciton line intensity for ∼40 QDs. In most cases, the intensity varies as the emission wavelength tunes continuously with the applied voltage. In Figure 2b, the *x*-polarized tuning curves (intensity vs wavelength) are summarized for all individual QDs, and the maximum intensity value within the ensemble of all recorded QDs shows a prominent peak at 728 nm (1.703 eV), matching very well the shape of the simulated enhancement spectrum shown in Figure 1b.

**Figure 2:**
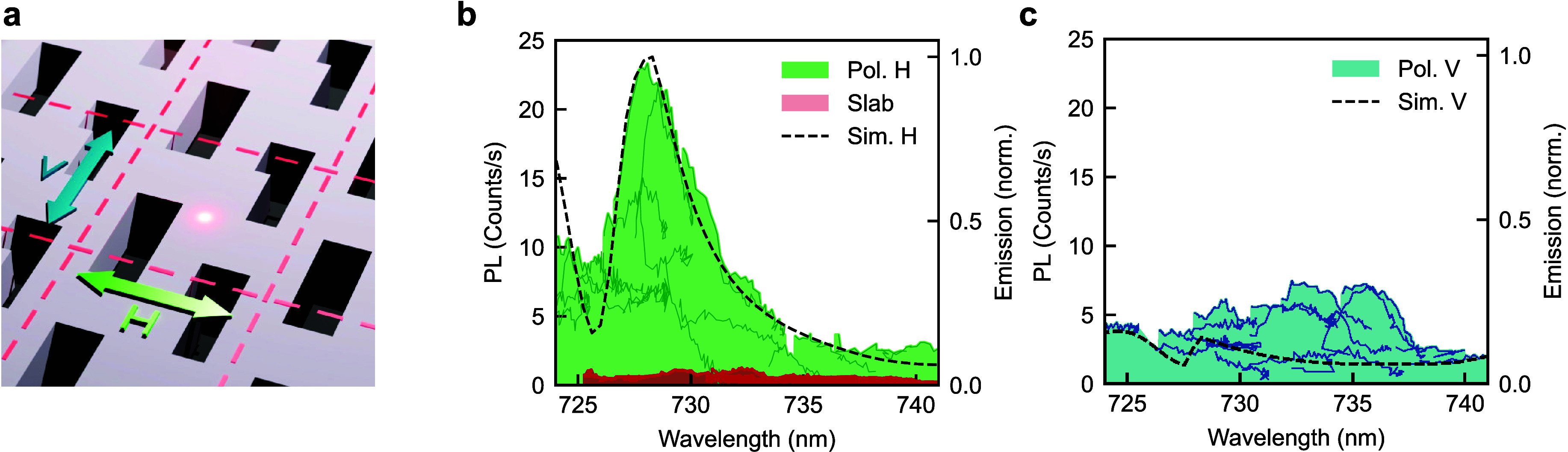
**a.** Illustration of the metasurface geometry with dashed lines marking the unit cell. **b, c.** PL tuning curves for metasurface-embedded QDs under bias for H-polarization (solid green lines in **b.**) and V-polarization (solid blue lines in **c.**). The shaded areas show the envelope of the maximum PL intensity over all of the QDs for each polarization. The red shaded area in **b.** shows the maximum PL envelope for QDs within an unpatterned area (slab) in the same sample for comparison.

We note that no single QD tunes over the entire 20 nm span shown in Figure 2b, and therefore, an ensemble of individual QDs is required to reveal the shape of the enhancement spectrum. While the PL intensity varies for different QDs, likely due to their random position in the *xy*-plane, the maximum intensity observed within the ensemble exceeds the maximum levels for QDs embedded in an unpatterned region (slab) adjacent to the metasurface by over one order of magnitude (red shaded area in Figure 2b). Furthermore, the orthogonally polarized (V) emission spectrum for the metasurface-embedded QDs shows a relatively uniform and lower-level emission enhancement over the same spectral range (Figure 2c). These polarization- and wavelength-dependent QD emission enhancement spectra confirm that the metasurface enhances photon outcoupling when the wavelength of QD emission is tuned into resonance with the two modes.

Having confirmed that the QD emission couples to the metasurface modes, we now explore whether multiple QDs within the same metasurface can be tuned to the same wavelength using the QCSE. First, we quantify the effect of applied bias. We observed the tuning of QD emission wavelength primarily in forward bias, in the range from ∼0.5 to ∼1.5 V, with no tuning in the range from ∼−1 to 0.5 V. This behavior can be understood after modeling the band structure using drift-diffusion and Poisson equations (COMSOL MultiPhysics) and determining the value of the electric field in the QD region (barrier). Under forward bias (Figure 3a), the applied voltage almost directly translates to the electric field *F* in the barrier, and that field tunes the QD exciton energy. In contrast, the bands in the barrier remain relatively flat for voltages from ∼−1 to 0.5 V due to the doped region near the Schottky barrier (Figure 3c). As a result, the electric field in the barrier remains low and relatively constant within the range of voltages. The flat-band conditions also correlate with generally higher PL levels (Figure 3b). At higher reverse bias voltages, the electric field in the barrier again increases, now in the opposite direction, leading to QD emission tuning. Under reverse bias, however, the QD emission intensity decreases significantly, likely due to excited charge carriers tunneling out of the QD (Figure 3c).

**Figure 3:**
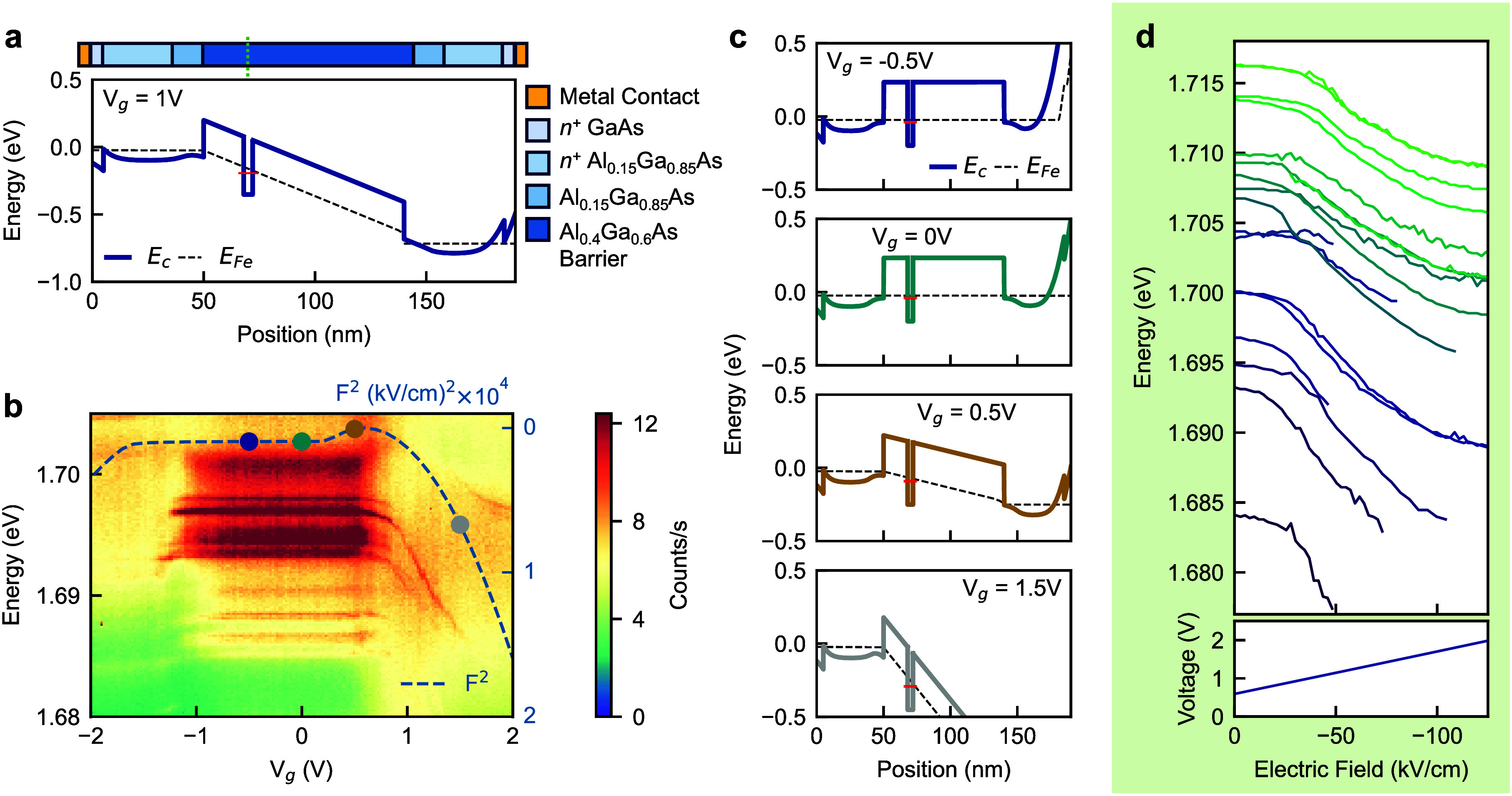
**a.** Numerically modeled conduction band profile of the heterostructure under a 1 V external bias. **b.** Experimental PL spectral map of QD tuning, with a square of the associated modeled electric field overlaid, explaining the tuning behavior. **c.** Conduction band profile under selected biases, showing the proportional band tilt under forward bias and a relatively flat band under reverse bias. **d.** Exciton energy tuning for several tested QDs within the metasurface plotted as a function of the applied electric field.

The band structure simulations allow us to extract the electric field *F* experienced by the QDs and plot the exciton energy as a function of the field (Figure 3d). The exciton peak shifts for each QD following approximately a quadratic function of the field (for *F* ranging from 0 to –50 kV/cm). However, the tuning varies slightly between individual QDs, suggesting that two QDs with different zero-field energies, *E*_0_ may tune to the same wavelength under the same applied field.

For quantitative analysis, we evaluated the dipole moment *p* and the polarizability β for each QD by fitting a quadratic function, *E*(*F*) = *E*_0_ – *pF* + *βF* ^2^ to the experimentally observed tuning curves (Figure 4a). The extracted values of *p* and β are plotted in Figures 4b and 4c, respectively. The relatively large variation in *p* indicates that excitons in a pair of QDs with similar *E*_0_ values could be tuned to exactly the same energy under the same applied voltage. The shaded regions in Figure 4b mark QDs that may potentially overlap spectrally under applied bias with a QD highlighted with a yellow circle. Assuming equal polarizability, the QD highlighted with a blue symbol would require a field of ∼−40 kV/cm to align spectrally with the yellow-highlighted QD. Another QD highlighted with a red symbol would require a field of ∼−100 kV/cm to align spectrally with the yellow-highlighted QD.

**Figure 4:**
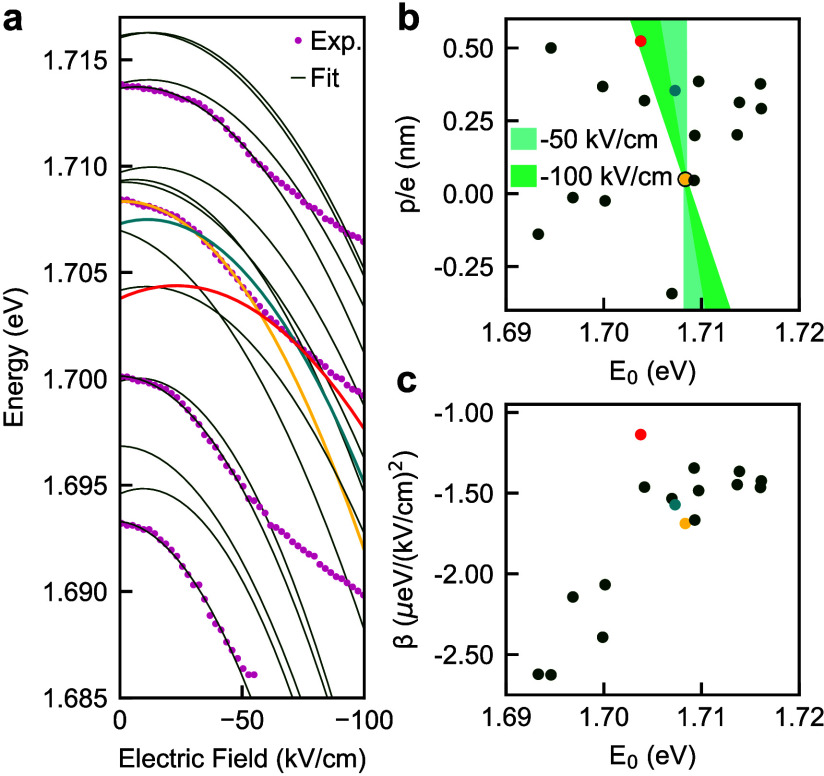
**a.** Models of the QD exciton energy tuning curves under applied field obtained by fittings of the quadratic function, *E*(*F*) = *E*_0_ – *pF* + *βF* ^2^, to the experimental data in Figure 3d, with four sets of experimental data demonstrating the close fit. **b, c.** Extracted dipole moment *p* and polarizability β for tested QDs. **b.** The shaded areas show parameter spaces within which QDs can be potentially aligned spectrally with the QD highlighted with a yellow circle using experimentally accessible values of the applied electric field.

For experimental verification, we selected a pair of QDs with similar *E*_0_, located within the metasurface only 6.6 μm apart yet sufficiently far from each other for collecting their individual spectra. Their PL tuning maps are plotted in Figures 5a,b with the tuning curves summarized in Figure 5c. The wavelength for the dominant exciton in QD1 tunes continuously with applied voltage, whereas for QD2, two exciton states are observed, both tuning continuously but only within narrower voltage ranges. Remarkably, the two exciton states of QD2 cross with the exciton tuning curve for QD1, one at a bias voltage of ∼0.675 V and the other at ∼0.925 V, as shown in Figure 5d together with selected spectra just before and just after the crossings. Another example of two QDs with overlapping exciton lines is shown in the Supporting Information. These results confirm experimentally that two QDs embedded in a nonlocal metasurface can produce photons at the same wavelength using an externally applied electric field. Furthermore, this is achieved while enhancing the QD outcoupling efficiency by over one order of magnitude.

**Figure 5:**
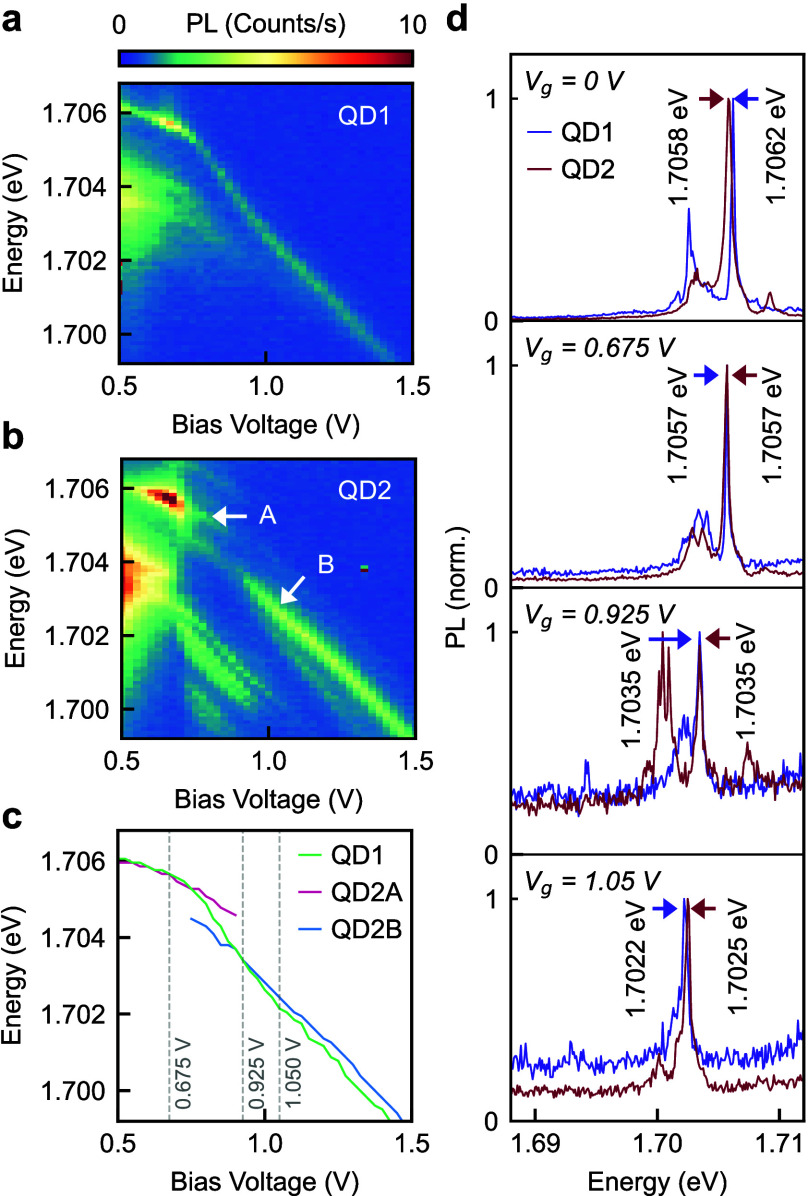
**a,b.** PL tuning maps for two metasurface-embedded QDs (QD1 and QD2) separated by 6.6 μm. Two exciton states in QD2 are labeled A and B. **c.** The tuning curves for QD1 and QD2 show two crossing points. **d.** Selected PL spectra of the two QDs showing their exciton emission lines at the same and different energies.

We demonstrate a nonlocal metasurface platform with embedded tunable GaAs LDE QDs coupled to an extended guided mode and showing enhanced free-space outcoupling. The tuning of the QD emission wavelength is realized via an applied external electrical bias, enabling continuous tuning of QD emission over 10–12 meV. While the QDs are not identical, and the same voltage is applied to all QDs within the metasurface simultaneously, the natural variation in the QD dipole moment allows us to tune two QDs into spectral alignment and in resonance with two overlapping modes in the nonlocal metasurface, one of which facilitates the photon outcoupling. As a result, two distant QDs can produce photons at the same wavelength with over 1 order of magnitude improved outcoupling efficiency to free space. This platform enables enhanced outcoupling without the need for precise placement of individual QDs, owing to the nonlocal and periodic nature of the modes. While this study only demonstrates the developed metasurface platform, further investigations of quantum properties of the embedded QDs, such as measurements of the second-order photon correlation function as well as photon cross-correlation measurements, will show the suitability of this platform for cooperative emission from sets of multiple quantum emitters and could open doors for quantum communication and information systems applications.

## Supplementary Material



## Notes

Additional detail regarding fabrication, design, modeling and characterization (PDF)
