# Supplementary material for: Voltage-Tunable Nonlocal Metasurface for Enhanced Outcoupling of Emission from Quantum Dots
Source: Nano Lett. 2026 Jan 20;26(10):3323–9. doi: 10.1021/acs.nanolett.5c04834 (PMC13003492; doi:10.1021/acs.nanolett.5c04834)
Supplement: Supplementary file 1 [file nl5c04834_si_001.pdf]

# Supplementary Information

## Voltage-Tunable Nonlocal Metasurface for Simultaneous Emission from Distant Quantum Dots

Samuel Prescott,[1] Prasad P. Iyer,[2,3] Sanghyeok Park, [2,3]
Stephanie Malek,[2,3] Jiho Noh, [2,3] Pingping Chen,[2,4] Chloe F. Doiron, [2,3]
Sadhvikas Addamane,[2,3] Igal Brener[2,3] and Oleg Mitrofanov[1,2]

1. *University College London, Electronic and Electrical Engineering, London WC1E 7JE, UK*
2. *Center for Integrated Nanotechnologies, Sandia National Laboratories, Albuquerque, New Mexico 87123, USA*
3. *Sandia National Laboratories, Albuquerque, New Mexico 87123, USA*
4. *University of Colorado Boulder, Electrical, Computer and Energy Engineering, Boulder, Colorado 80309, USA*

**Table of contents:**

**S1. Fabrication of Metasurfaces with Embedded GaAs Quantum Dots**
**S2. Metasurface Design**
**S3. Electronic Band Structures**
**S4. Tuning of Quantum Dot Emission Wavelength**
**S5. Location of Spectrally-aligned Paired Quantum Dots**

## S1. Fabrication of Metasurfaces with Embedded GaAs Quantum Dots

### *Growth of GaAs quantum dots in n-i-n heterostructure*

Local-droplet-etched (LDE) GaAs QDs were grown by molecular beam epitaxy (MBE) within an $Al_{0.4}Ga_{0.6}As$ barrier, on a (100) GaAs substrate (wafer number VB1667). The barrier was sandwiched in between two doped layers forming a vertical *n-i-n* stack. The full MBE growth design and parameters are summarized in Table S1 and are displayed schematically in Fig. S1.

| | | QD sample design and MBE growth parameters | | |
|---|---|---|---|---|
| **Layer #** | **Material** | **Thickness** | **Doping** | **Description** |
| 1 | GaAs | 5 nm | Si ($n$), $2.35 \times 10^{18}$ cm$^{-3}$ | Doped layer |
| 2 | $Al_{0.15}Ga_{0.85}As$ | 35 nm | Si ($n$), $2 \times 10^{18}$ cm$^{-3}$ | Doped layer |
| 3 | $Al_{0.15}Ga_{0.85}As$ | 10 nm | - | Interlayer |
| 4 | $Al_{0.4}Ga_{0.6}As$ | 20 nm | - | Barrier |
| 5 | GaAs | 1.7 nm | - | QD (grown using migration enhanced epitaxy) |
| - | - | - | - | Droplet etching |
| - | Al | 0.6 ML | - | Droplet formation |
| 6 | $Al_{0.4}Ga_{0.6}As$ | 70 nm | - | Barrier |
| 7 | $Al_{0.15}Ga_{0.85}As$ | 10 nm | - | Interlayer |
| 8 | $Al_{0.15}Ga_{0.85}As$ | 35 nm | Si ($n$), $2 \times 10^{18}$ cm$^{-3}$ | Doped layer |
| 9 | GaAs | 5 nm | Si ($n$), $2.35 \times 10^{18}$ cm$^{-3}$ | Doped layer |
| 10 | $Al_{0.75}Ga_{0.25}As$ | 500 nm | - | Sacrificial layer |
| 11 | GaAs | 200 nm | - | Smoothing layer |
| 12 | GaAs (100) | | | Substrate |

**Table S1.** LDE GaAs QD sample design and MBE growth parameters.

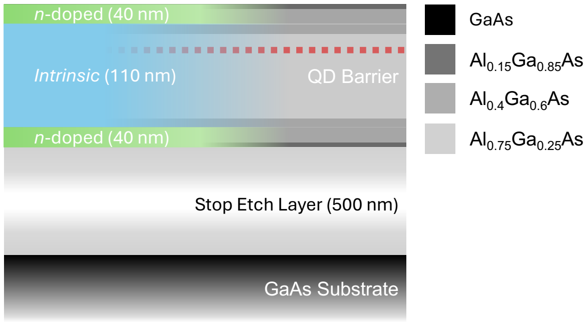


**Fig. S1.** Schematic design of the layer stack, showing the *n-i-n* structure on top of sacrificial layers.

*Fabrication of metasurfaces with embedded tunable quantum dots*

Five devices, each containing a 254 x 666 μm$^2$ mesa with four 120 x 120 μm$^2$ metasurfaces, were fabricated on a 10 x 10 mm$^2$ sample. The metasurfaces were patterned on top of the sample surface using electron beam lithography (accelerating voltage: 100 kV, beam current: 500 pA, dose: 420 μC/cm$^2$ with 105-region proximity effect correction) with a bi-layer ZEP 520 A on PMMA 950 A2 resist combination (spun at 5000 RPM and baked at 180°C for 90 s). The exposed pattern was developed in chilled n-amyl acetate (cooled in a sealed container in a -5°C water bath for two hours before development) for 180 s (remaining in the chilled water bath during development, with a loose-fitting lid). The metasurface pattern was then vertically etched approximately 190 nm using chlorine reactive ion etching (RIE). The resist was then weakened by oxygen plasma etching (120 s) and removed using Remover PG at 85°C for 2 h.

The first metal contact was defined using photolithography (photoresist: AZ 5214), with a metal stack (60 nm AuGe (88:12)/10 nm Ni/60 nm AuGe (88:12)) deposited using electron beam evaporation. After deposition, the remaining photoresist was removed from the non-contact area through a lift-off process using Remover PG at 85°C for 2 h. The contact was then annealed at 380°C for 60 s. The five devices on the sample were electrically isolated from each other, by performing a second RIE step to etch the wafer stack in between each device down through the lower doped layers.

The sample was epoxy-bonded to a sapphire plate (patterned side facing the sapphire), and the GaAs substrate was removed using lapping and wet etching in a citric acid solution (100 ml water, 100 g citric acid (monohydrate), 32.5 ml $H_2O_2$), stopping at the $Al_{0.75}Ga_{0.25}As$ layer (Layer 10, Table S1). The stop-etch layer was then etched away using a hydrochloric acid solution (20 ml water, 20 ml HCl) revealing the n-doped GaAs layer (Layer 9, Table S1). The second contact was deposited on the now exposed GaAs layer by repeating the same metal evaporation process as for the first contact; and without the annealing, the metal formed a Schottky junction with the semiconductor.

To reveal the first contact, photoresist was used to cover and protect the metasurfaces and the Schottky contact. Then the remaining wafer stack was etched using a phosphoric acid solution (200 ml water, 20 ml $H_3PO_4$, 4 ml $H_2O_2$). Finally, gold wires were soldered to the contact pads using indium solder for photoluminescence measurements under bias. A schematic cross-section of the device is shown in Fig. S2.

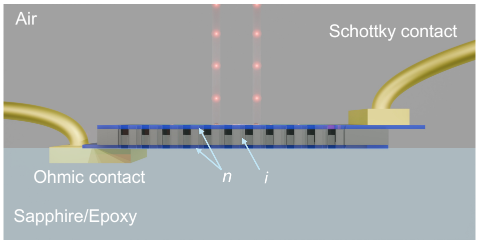


**Fig. S2.** Device cross section, showing the contacts and *n-i-n* metasurface structure.

## S2. Metasurface Design

Fig. S3 illustrates the development of metasurface design, starting from a dimer unit cell (Fig. S3a). The first row shows the progression of the unit cell design, with the first perturbation introduced through a vertical offset between the two holes (first three columns) and the second perturbation introduced by adding a tab to one of the holes (final column).

### *QD emission outcoupling*

Spatial profiles of two modes, A (radiative mode) and B (guided mode), are shown in the second and third rows Fig. S3b,c, for the respective unit cell designs in the first row Fig. S3a (aligned by columns). The spatial profiles were determined using FDTD simulations, with the periodic boundary conditions and plane wave excitation normal to the surface.

For the dimer design (first column), the profiles of the two modes vary significantly: mode A is distributed mostly in the holes, while mode B is distributed in the dielectric, along the vertical bars. As the vertical offset along the $y$-axis is introduced, the modes progressively become similar in spatial distribution (A3 compared to B3), with mode A still having the maximum electric field within the holes. At this point, modes A and B are also still spectrally separated by 14 nm, as can be seen in Fig. S3d, showing a map of reflection spectra obtained from the FDTD simulations for a range of perturbations. The selected hole offset perturbation is marked by a horizontal black dashed line in the map. To shift mode A's wavelength to that of mode B, a tab is introduced to the right-hand hole (final column of Fig. S3a,b,c). The tap leads to a good spatial overlap between the field distributions for modes A and B, as well as spectral alignment.

To investigate the effect of metasurface on photon outcoupling, we simulated the outcoupling efficiency for an $x$-polarized point dipole emitter, placed at the position marked by the red dot in the unit cells in Fig. S3a (FDTD simulations, periodic boundary conditions). The emission results are shown in Fig. S3e for the same hole offset and tab size sweeps. At a tab width of $a/P = 0.085$, the map displays the two modes overlap, resulting in a relatively narrow emission peak, which combines the narrow linewidth of mode B and the enhanced outcoupling of mode A (the red dashed line in Fig. S3e). The mode overlap results in a distinctive spectral feature with an ~5 nm wide peak in enhancement.

### *QD emission coupling to extended mode*

Next, we consider properties of mode B and its role in potential coupling between multiple QDs. We simulated the electric field distribution in a metasurface consisting of 11 x 11 unit cells for an $x$-axis polarized (H) dipole source placed in the central cell, at the position marked by the red dot in Fig. S3a (final column), the location of the highest electric field magnitude for Mode B.

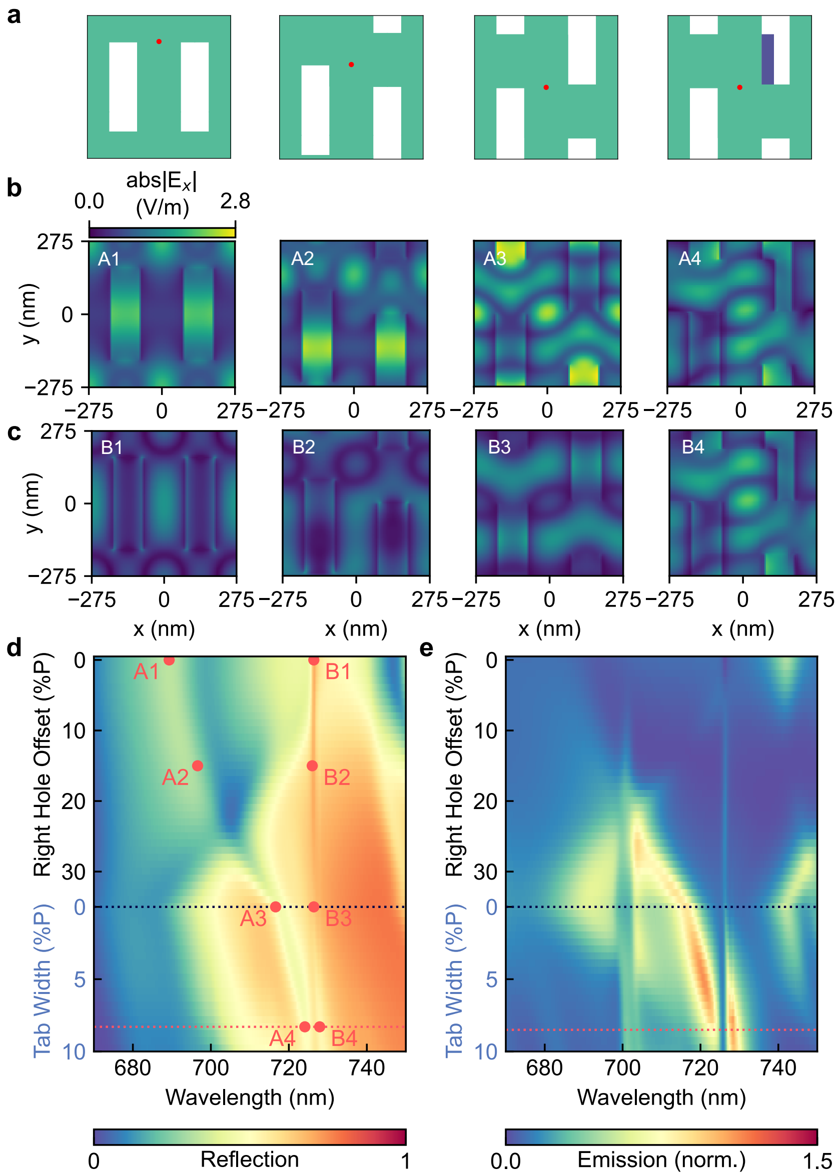


**Fig. S3.** Metasurface design development illustrating evolution of two modes: mode A is the radiative mode, whereas mode B is a higher Q-factor guided mode. **a.** Four stages of the unit cell design, with colors representing different materials: green – dielectric; white – holes, filled by epoxy; blue – perturbation (dielectric); red dots show the point dipole location in the simulations. **b,c.** Electric field distribution for Mode A (**b.**), and Mode B (**c.**) for each of the unit cells in **a. d.** Normal incidence reflection map showing the spectrum evolution as the unit cell progresses through the designs shown in **a.** Designs in each column are marked as A1-A4 and B1-B4 in **d. e.** Simulated emission map for a point dipole source (*x*-polarized) located at the red dot in **a.** for the same sweeps of metasurface parameters as in **d.**

In these simulations, perfectly matched layer (PML) boundary conditions are used on all sides. Figure S4 shows the emission from the point dipole spreads out primarily along the *y*-axis, indicating that the emission couples to the guided mode of the metasurface. The field spreads out over 3 μm away from the source, suggesting that two emitters separated by this distance may exhibit cooperative emission. This effect is present for wide range of wavelengths, however the enhanced outcoupling shown in Fig. 3e occurs in a much narrower range (~5 nm) with a peak emission peak (728 nm). We note that it was not possible to simulate a larger area metasurface compared to the size shown in Fig. S4 due to resource limitations.

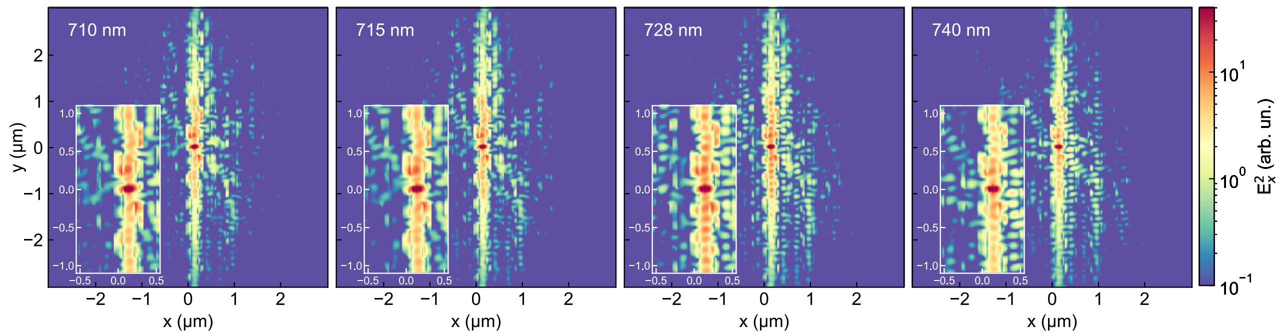


**Fig. S4.** Simulated electric field intensity, $E_x^2$, due to the emission from a *x*-polarized (H) dipole source in the 11 x 11 cells finite-area metasurface. *Insets* show a magnified central region of the metasurface.

### *Far-field emission patterns*

To further illustrate the effect of metasurface modes on emission outcoupling, we modelled the far-field emission pattern for a QD embedded in the metasurface. We used finite-area simulations with 11 x 11 metasurface unit cells and an x-polarized dipole emitter (aligned to the φ = 0° direction in Fig. S5), surrounded by PML absorbing boundary conditions.

For the selected design (B), the emission pattern at the wavelength corresponding to the overlap of the radiative and guided modes (728 nm), the far-field emission pattern displays a narrow lobe along the surface normal and it is confined within an angle θ of 10 degrees. In contrast, the same metasurface exhibits no prominent lobes at a slightly shifted wavelength of 735 nm. Similarly, at 728 nm, where only the guided mode is present for metasurface design A (no coupling to the radiative mode), the outcoupling is poor and it lacks the directionality of design B.

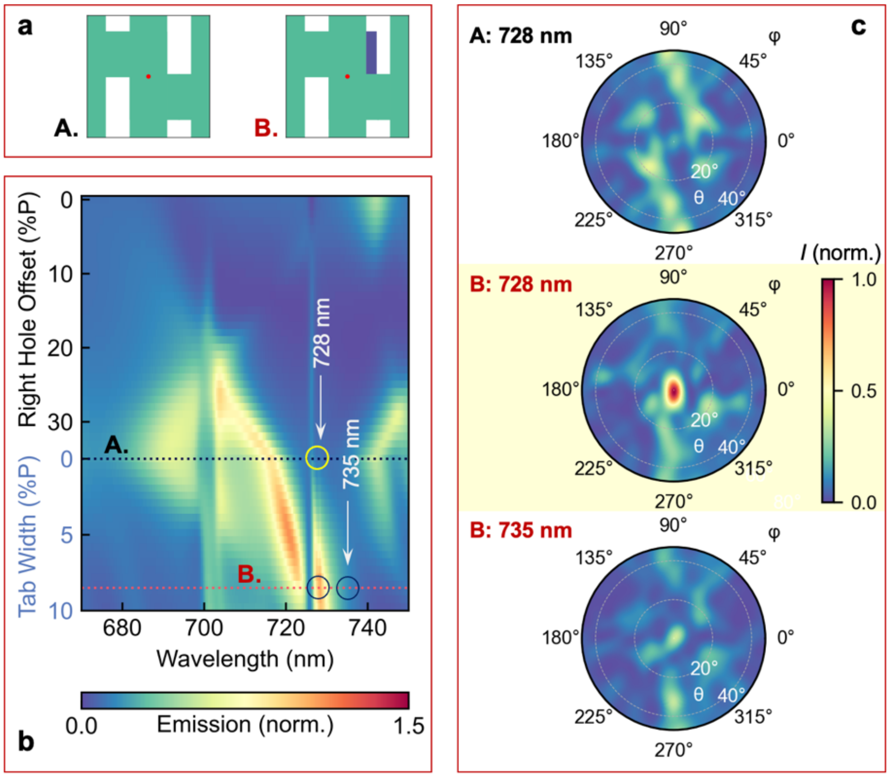


**Fig. S5.** Far-field emission patterns of selected metasurface designs A and B. a. Metasurface unit cell diagrams for designs A and B. b. Emission spectra for a QD embedded in metasurfaces of different designs (periodic simulations, reproduced from Fig. S3e) showing the radiative and guided mode overlapping at ~728 nm. Far-field emission patterns calculated using the finite-area (11 x 11 unit cells) simulations, for three cases marked in (b): Metasurface A at 728 nm (top), Metasurface B at 728nm (center) and Metasurface B at 735 nm.

## *Metasurface design*

Fig. S6 shows the selected fabricated metasurface unit cell design with dimensions in absolute units (nm) and relative to the period *P*.

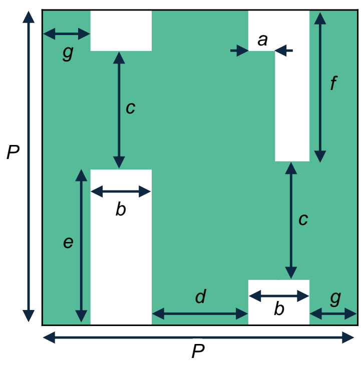


**Fig. S6.** Metasurface unit cell design with green areas showing the dielectric and white areas showing the epoxy.

## *Periodicity adjustment process*

Adjusting the y-axis period in the metasurface shifts the guided mode to a greater extent than the radiative mode (Fig. S7). We optimized the y-axis period together with the right hole perturbation tab size to align the two modes at a desired wavelength of ~728 nm.

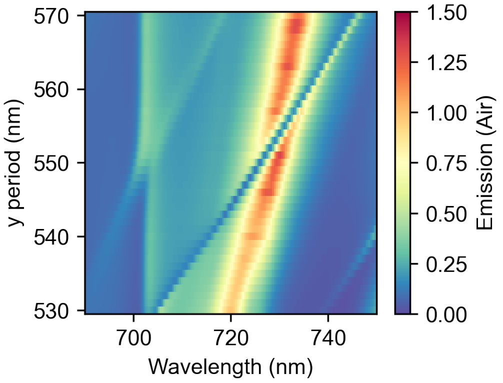


**Fig. S7.** The effect of variation in the y-axis period on the modelled dipole emission from the metasurface.

## S3. Electronic Band Structure

The electronic structure of the device was modelled using COMSOL MultiPhysics' drift-diffusion and Poisson equation solvers. The conduction band profile was simulated for a range of applied biases and selected profiles are shown in Fig. S8.

For illustration purposes, the electron energy level for a typical QD was added as a red line in each profile. The QD exciton energy was assumed to be equal to the experimentally determined emission energy for a typical QD (~1.7 eV). The confining energies for the electron and the hole in the QD were weighted using the corresponding effective masses in GaAs.

Figure S8 shows that in forward bias, the conduction band in the barrier region tilts resulting in the electric field experienced by the QD. The electric field value in the barrier for all applied voltages was calculated directly from these simulations and used in the Article. The profiles for the reverse bias voltages show that the applied voltage is dropped almost entirely across the Schottky barrier region (right hand side of the structure), explaining the lack of tuning in reverse bias.

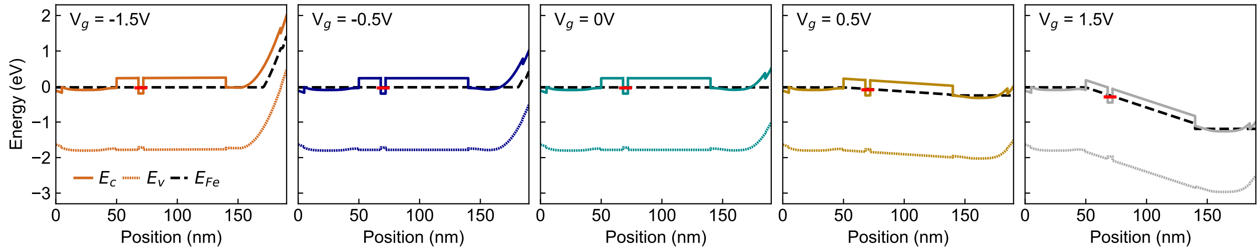


**Fig. S8.** Conduction and valence band profiles for five applied bias voltages (-1.5 V, -0.5 V, 0 V, 0.5 V, and 1.5 V), showing the conduction band edge (colored solid lines), Fermi level (black dashed line), valence band edge (colored dotted lines), and the electron level in the QD (red bar).

## S4. Photoluminescence Spectroscopy of Quantum Dots Embedded in Metasurface

The sample was mounted in a cryostat on x/y/z piezoelectric stages, with electrical connections soldered to the metasurface contacts and passed over the cryostat feedthroughs to a multimeter, SourceMeter Unit (SMU), Kiethly 2420, for applying a bias and monitoring the current. A 40X aspherical objective lens was placed within the cryostat for excitation and collection of QD photoluminescence (PL).

The excitation was realized using a 516 nm laser entering the cryostat through an optical port and focused on the sample surface by the objective lens. PL from the sample was collected by the same lens and split off from the excitation path using a dichroic mirror. Additional filtering of the collected light was performed using a 600 nm long-pass filter and a linear polarizer (to independently measure orthogonal polarization components of the PL). The excitation beam position on the sample was monitored using a removable beamsplitter and a digital camera. Figure S9 shows the locations of the 28 measured metasurface-embedded QDs spread throughout the metasurface. We note that the beam splitter was removed during spectral acquisition. The PL was then coupled into a single-mode fiber and guided to a 320 mm length grating spectrometer equipped with a 1200 groove per mm grating and a thermoelectrically-cooled CCD detector array (PIXIS 100) for spectral analysis.

For each QD, we adjusted the sample position to align the QD with the excitation beam using the piezoelectric stages and the monitoring camera. The sample's contacts were grounded during the alignment. Then the contacts were connected to SMU to apply the bias while the current through the sample was monitored. PL spectra were measured for both polarizations using the spectrometer (acquisition time 60 s) for each step in a voltage sweep across a range from -2.0 V to 2.0 V. The results were plotted as 2D color maps (photon energy vs. bias voltage) with color representing the spectrometer count rate.

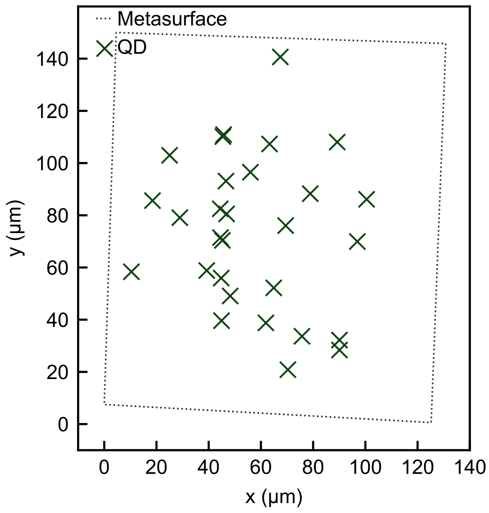


**Fig. S9**. Location of measured QDs within the metasurface area (outlined by the dotted line).

Figure S10 shows polarization-resolved PL spectra for the 28 QDs over a voltage range of 0.5 V to 2 V, with the H- and V-polarizations displayed in Fig. S10a and Fig. S10b, respectively. These spectra are plotted to show the shift of the primary exciton line at zero bias, $E_0$. The common background PL was subtracted from the spectra. The exciton energy and the peak intensity were then extracted for each QD and combined in Fig. 2 of the Article.

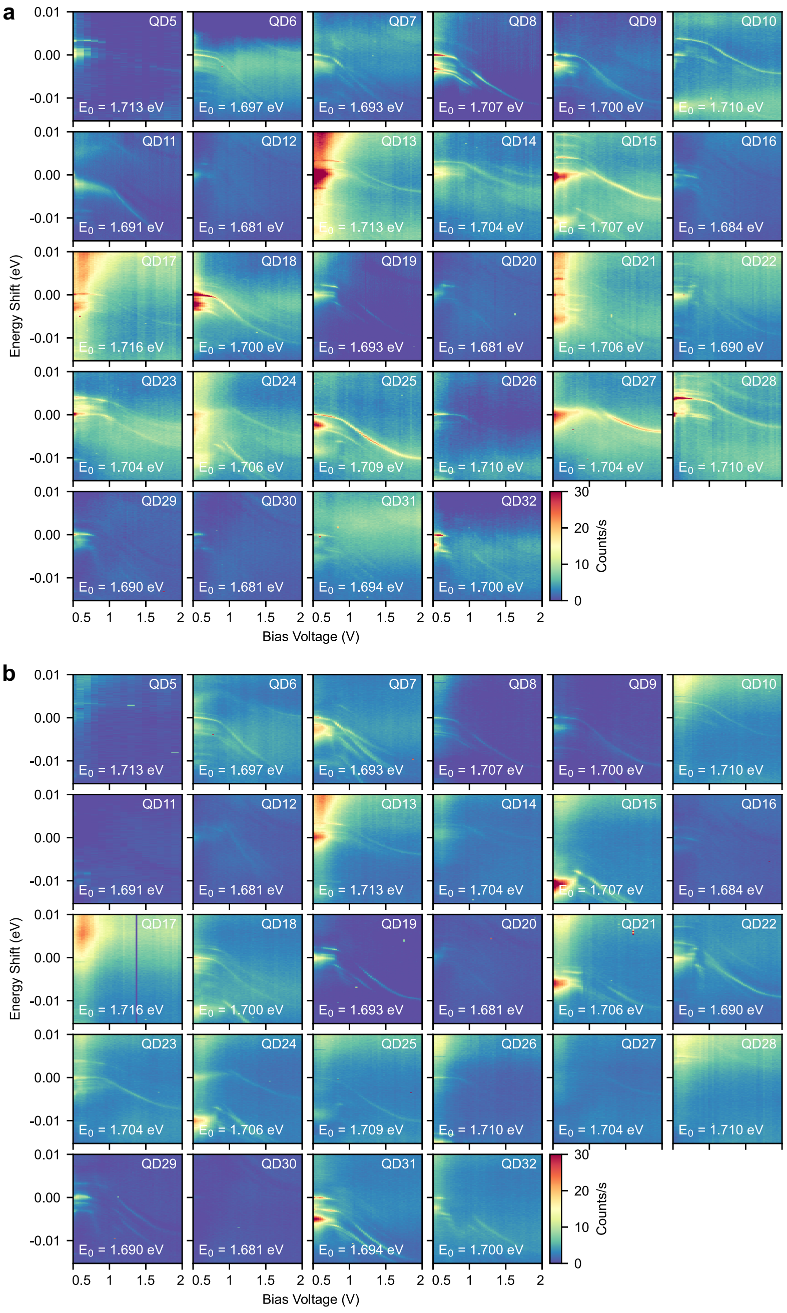


**Fig. S10. a.** H-polarized, and **b.** V-polarized, PL spectra of 28 QDs within the metasurface, showing the voltage-dependent shift in exciton energy.

## S5. Location of Spectrally-aligned Paired Quantum Dots

### *QD1 and QD2*

Figure S11 shows the relative position of the two QDs presented in Fig. 5 of the Article. The QDs are separated by ~6.6 μm, mainly along the *y*-axis of the metasurface, the axis of guided mode shown in Fig. S4.

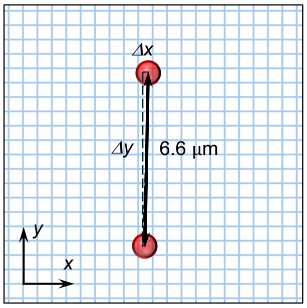


**Fig. S11.** Relative position of QD1 and QD2 (Fig. 5, Article), $\Delta x = 0.39$ μm, $\Delta y = 6.6$ μm.

### *QD3 and QD4*

Figure S12 shows a second pair of QDs, which can be tuned to align spectrally as shown in Fig. S12d. The QDs are separated by ~6.8 μm.

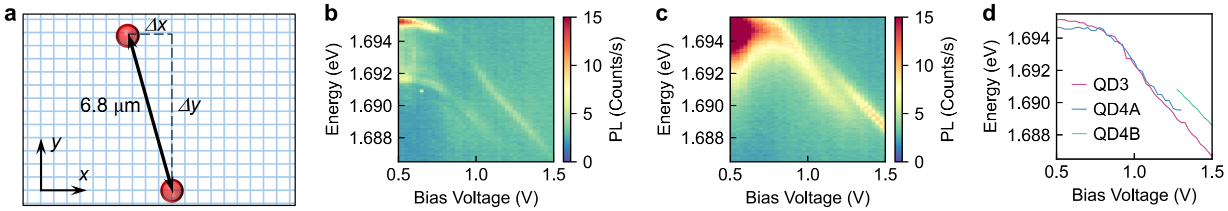


**Fig. S12.** Tuning of the second pair of QDs. **a.** Relative position of QD3 and QD4, $\Delta x =$ 1.9 μm, $\Delta y = 6.5$ μm. **b.** PL tuning map of QD3. **c.** PL tuning map of QD4. **d.** Extracted exciton energies of QD3 and QD4, with two exciton states in QD4 labelled as QD4A and QD4B.
